# *CCR2*/CCL2 and *CMKLR1*/RvE1 chemokines system levels are associated with insulin resistance in rheumatoid arthritis

**DOI:** 10.1371/journal.pone.0246054

**Published:** 2021-01-28

**Authors:** Gustavo Ignacio Diaz-Rubio, Fernanda-Isadora Corona-Meraz, Perla-Monserrat Madrigal-Ruiz, Jesús-Aureliano Robles-De Anda, Eduardo Gómez-Bañuelos, Jorge Castro-Albarran, Luis-Javier Flores-Alvarado, Mónica Vázquez-Del Mercado, Felipe de Jesús Pérez-Vázquez, Oscar-Enrique Pizano-Martínez, Rosa-Elena Navarro-Hernández

**Affiliations:** 1 Doctorado en Ciencias en Biología Molecular en Medicina, Departamento de Biología Molecular y Genómica, Centro Universitario de Ciencias de la Salud, Universidad de Guadalajara, Guadalajara, Jalisco, México; 2 UDG-CA-701, Inmunometabolismo en Enfermedades Complejas y Envejecimiento, Departamento de Biología Molecular y Genómica, Centro Universitario de Ciencias de la Salud, Universidad de Guadalajara, Guadalajara, Jalisco, México; 3 Departamento de Ciencias Biomédicas, División de Ciencias de la Salud, Centro Universitario de Tonalá, Tonalá, Jalisco, México; 4 Departamento de Biología Molecular y Genómica, Centro Universitario de Ciencias de la Salud, Universidad de Guadalajara, Guadalajara, Jalisco, México; 5 Division of Rheumatology, The Johns Hopkins University School of Medicine, Baltimore, Maryland, United States of America; 6 Departamento de Ciencias de la Salud y Ecología Humana, División de Desarrollo Regional, Centro Universitario de la Costa Sur, Autlán de Navarro, Jalisco, México; 7 Instituto de Investigación en Reumatología y del Sistema Músculo Esquelético, Departamento de Biología Molecular y Genómica, Centro Universitario de Ciencias de la Salud, Universidad de Guadalajara, Guadalajara, Jalisco, México; 8 Servicio de Reumatología, División de Medicina Interna, Hospital Civil Dr. Juan I. Menchaca, Guadalajara, Jalisco, México; Virgen Macarena University Hospital, School of Medicine, University of Seville, SPAIN

## Abstract

Rheumatoid arthritis (RA) has been associated with insulin resistance (IR). Due to an excess in storage of white adipose tissue, IR has an inflammatory process that overlaps with RA. This is performed by the activation/migration of monocytes carried out by the CCR2/CCL2 and CMKLR1/RvE1 chemokines systems. Furthermore, these can potentiate chronic inflammation which is the central axis in the immunopathogenesis of RA. We evaluated the association between the relative expression of *CCR2* and *CMKLR1* and the serum levels of their ligands CCL2 and RvE1, in the context of adiposity status with IR as a comorbidity in RA. We studied 138 controls and 138 RA-patients classified with and without IR. We evaluated adiposity, RA activity, IR status and immunometabolic profiles by routine methods. Insulin, CCL2 and RvE1 serum levels were determined by ELISA. Relative expression of *CCR2*, *CMKLR1* and *RPS28* as constitutive gene by SYBR green RT-qPCR and 2^-ΔΔC^_T_ method. Increased measurements were observed of body adiposity and metabolic status as follows: RA with IR>control group with IR>RA without IR> control group without IR. *CCR2* and *CMKLR1* relative expression was increased in RA without IR versus control without IR. *CCR2*: 2.3- and 1.3-fold increase and *CMKLR1*: 3.5- and 2.7-fold increase, respectively. Whereas, *CCR2* expression correlates with *CMKLR1* expression (*rho* = 0.331) and IR status (*rho* = 0.497 to 0.548). *CMKLR1* expression correlates with inflammation markers (*rho* = 0.224 to 0.418). CCL2 levels were increased in the RA groups but levels of RvE1 were increased in RA without IR. We conclude that in RA with IR, the chemokine receptors expression pattern showed a parallel increase with their respective ligands. RA and IR in conjunction with the pathological distribution of body fat mass might exacerbate chronic inflammation. These results suggest that high CCL2 levels and compensatory RvE1 levels might not be enough to resolve the inflammation by themselves.

## Introduction

Rheumatoid arthritis (RA) is an inflammatory disease that develops between the fourth and seventh decades of life, with a worldwide prevalence of 1%. In its clinical course the most evident pathological manifestation is joint damage shown by synovitis, progressive degradation of articular cartilage and erosion of the subchondral bone. An early event in the joint damage mechanism is the extravasation of leukocytes to the intra-articular space which is due to the expression of chemokines systems [1, 2].

Furthermore, the inflammatory response is the central axis in the immuno-pathogenesis in RA [3, 4]. The early extravasation of leukocytes results in a redundant production of chemokines and pro-inflammatory cytokines that exacerbate the inflammation. This sustained state eventually promotes the characteristic formation of *pannus* which confirms the established pathological process of RA in the body [5].

RA, as a systemic inflammatory disease, during its clinical progression has been associated with comorbidities such as insulin resistance (IR) [6–8] with alterations in relation to the body´s pathologic redistribution of white adipose tissue (WAT) and the rise of immuno-metabolic markers levels [9, 10].

Insulin resistance is defined as the ineffectiveness of insulin to reduce blood glucose levels [11]. The first predisposing factor is the excess in storage of WAT with pathological distribution in the body, which generates the development of chronic low-grade subclinical inflammation [12]. This process increases the migration of monocytes from the blood circulation to WAT. Transmigrated monocytes polarize towards M1 phenotype which produce pro-inflammatory chemokines and cytokines that deregulate lipid metabolism in a redundant process [13].

Therefore, the common denominator between RA and IR, is systemic inflammation, supported by the increased levels of pro-inflammatory cytokines along with a redundant effect on the cells of the parenchyma of WAT and joint capsule with a gradient of clinical manifestation [14].

The foremost chemokines systems which are key for the activation of the inflammatory process are: the C-C chemokine receptor type 2 (CCR2), chemerin receptor 1 (Chemerin 1, also identified as Chemerin Chemokine-Like Receptor 1, CMKLR1); as well as its ligands: C-C motif chemokine ligand 2 (CCL2, previously named MCP-1) and resolvin E1 (RvE1), respectively [15, 16].

Inside of WAT, the CCR2/CCL2 axis induces adipocyte differentiation; in addition, it has been shown that the increase in its expression is regulated by pro-inflammatory cytokines, that in response also induce IR in adipocytes. In contrast, the secretion of CCL2 from adipocytes can be down-regulated by adiponectin, IL-4 and IL-10 which in turn prevent IR [17, 18]. Whereas, CMKLR1 is expressed in monocytes and macrophages and upregulated in M1 macrophages. It is reported that the interaction of the components of the CMKLR1/RvE1 axis activates the resolution process of acute or chronic inflammation, first by the recruitment of monocytes/macrophages to inflamed tissues and then by continuing the polarization of M2 phenotype. In particular, RvE1 is the second ligand of CMKLR1 which comes from the eicosapentaenoic acid (EPA) present in the diet, and its favorable properties under conditions associated with chronic inflammation [15].

In the RA patients with IR as a comorbidity, the relative expression levels of *CCR2* and *CMKLR1* and serum levels of their respective ligands (CCL2 and RvE1) has not been evaluated in the context of pathological distribution of the body adipose tissue.

## Materials and methods

A cross-sectional study was conducted in RA patients (n = 138) aged 18 to 63 years. Ethical appropriateness and biosecurity guidelines were ensured based on the principles of the Declaration of Helsinki [19]. The study group recruitment was conducted under consent and authorization from Institutional Boards Committees of Hospital Civil ‘‘Dr. Juan I. Menchaca” and the institution (Universidad de Guadalajara). Before enrolment, patients were informed about all implications of participating in the study, after which they agreed to and signed a consent form.

### Study group

#### Control group

Adult volunteers (n = 138) were recruited from the general population and were matched by sex, age (± 2 years) and body mass index (BMI, according with World Health Organization classification (WHO) criteria, i.e. normal weight, pre-obesity and obesity) with RA patients.

Inclusion criteria for the study were considered as follows: individuals who at the time of the study did not present, infectious diseases, hypertension, pregnancy, anemia, diagnosis of cardiovascular disease, malignancy, and renal or metabolic diseases such as type 2 diabetes (T2D). Subjects with a history of tobacco or drug use or under any medication were excluded. All subjects underwent a clinical examination to confirm a clinical healthy condition and a stable weight for the last three months.

#### Rheumatoid arthritis group

RA patients were recruited from an outpatient rheumatology service of a tertiary referral hospital in Guadalajara, México, who met the 1987 American College of Rheumatology and 2010 European League Against Rheumatism criteria [20]. Twelve percent of these patients had positive history of tobacco use, however, none of them were current smokers at the time of the study. All RA patients were treated with synthetic disease-modifying anti-rheumatic drugs (DMARDs) as, monotherapy (methotrexate or Sulfasalazine), or in combination, i.e. double (Methotrexate + Chloroquine or Methotrexate + Sulfasalazine or Chloroquine + Sulfasalazine), or triple (Methotrexate + Chloroquine + Sulfasalazine) therapy. We excluded patients under treatment with corticosteroids, and/or anti-depressants’ drugs or with known comorbidities (T2D, hypertension, thyroid dysfunction and renal or liver disease).

### Main outcome variable

Participants were classified with IR per Stern criteria [21] as follows: when the homeostasis model assessment (HOMA)-IR was higher than 4.65 or the body mass index (BMI) higher than 27.5 kg/m^2^ and a HOMA-IR higher than 3.6.

### Procedures

#### Subjects’ clinical assessments

Each control subject and RA patient was interviewed by a medical and nutritionist staff using structured questionnaires to gather demographic and nutritional evaluation data, as well as family medical history and the use of any medication. General physical examination and clinical assessment were performed by a rheumatologist and who also evaluated the RA patient’s disease activity using DAS28-CRP (disease activity score on 28 joints with levels of C-reactive protein) [22], with higher values meaning a higher disease activity thereby classifying its activity in 4 categories: remission ≤2.6, low 2.6–3.2, moderate 3.2–5.1, and high > 5.1 [23].

#### Evaluation of body adiposity status in all study subjects

Measurements of body composition: (body weight (kg), total body non-fat and fat mass: total, trunk and upper and lower limbs [relative (%) and absolute (kg)]) were determined by bioelectrical impedance analysis, (TANITA BC-418 segmental body composition analyser. Tokyo, JPN) to the nearest 0.1 kg.

Five skinfold thickness measurements (abdominal, bicipital, tricipital, subscapular and suprailiac) were obtained on the right side of the body by using a Harpenden skinfold caliper (opened to 80 mm with a precision of ± 0.2 mm, and a constant pressure of 10 g/mm^2^; Holtain Ltd. Croswell, Crymych, Pembs, SA41 3UF, UK), in accordance with the recommended procedures [24, 25].

Body dimensions were measured as follows (cm): height was measured to the nearest milimeter by using a stadiometer (Seca GmbH & Co. KG. Hamburg, Germany); waist circumference (WC), hip circumference (HC) and coronal abdominal diameter, were measured with an anthropometric steel tape (GULICK® length 0–180 cm precision ± 0.1; USA). Sagittal abdominal diameter was measured at the level of the iliac crest (L4 –L5) using an abdominal caliper (precision ± 0.1 cm, Holtain Ltd. Croswell, Crymych, Pembs, SA41 3UF, UK.) [26, 27].

All of the values of the anthropometric indicators were performed by the same nutritionist in duplicate for each subject. In addition to the BMI and body fat ratio, other abdominal adiposity indexes were calculated [28–31]:

**Table:** 

Index	Equation
BMI (kg/m^2^)	= body weight (kg)/[height (m)]^2^
Body fat ratio (kg/m^2^)	= total body fat mass (kg)/[height (m)]^2^
Waist to height ratio	= WC (cm) /height (cm)
Waist-hip ratio	= WC (cm) /HC (cm)
Visceral area (cm^2^)	= π (WC/2π−abdominal skinfold)^2^
Abdominal volume index (L)	= [2(WC^2^ + 0.7 (WC–HC)^2^]/1000
Visceral adiposity index	= (WC/36.58 + (1.896*BMI)) 6 (TG/0.81) 6 (1.52/HDLc).

#### Blood samples from study subjects

After confirmed fasting for the previous 12 hours for all study subjects, we obtained three venous blood samples, with and without anticoagulant [1) Sedisystem tube Cat. 366676, 2) PAXgene Blood RNA Tube Cat. 762165 and 3) BD SST Plus −13mm CAT. 368159, BD Diagnostic Systems Montenegro 1402 (C1427AND), Buenos Aires, Argentina]. Samples without anticoagulant were allowed to clot for 30 minutes at 20°C before centrifugation for 10 minutes/20°C at 1509 RCF (Rotanta 460R, Andreas Hettich GmbH & Co. KG.); serum was collected and stored immediately at −20°C until further analysis of ELISA proteins, inflammatory and metabolic markers.

#### Subjects’ immuno-metabolic profile

Disease indicators and metabolic marker levels, along with enzymatic and immuno-turbidimetry assays (Randox Laboratories; 55 Diamond Road, Crumlin Co. Antrim, Northern Ireland, UK) were performed to quantify basal serum of albumin (g/dL), aspartate aminotransferase (U/L), rheumatoid factor (IU/mL), glucose, and lipid profile (mg/dL) [*i*.*e*. triglycerides, total cholesterol, high (HDLc) and low (LDLc) density lipoprotein cholesterol, apolipoproteins A-1 (Apo A-1) and B (Apo B)]. Also very low density lipoprotein cholesterol (VLDLc) was calculated using the Friedewald formula [32]. Based on the lipid profile measurements we calculated the following cholesterol ratios: triglycerides/HDLc, total cholesterol/HDLc, LDLc/HDLc, and Apo B/Apo A-1.

High-sensitivity C-reactive protein (CRP with a limit of detection of 0.15 mg/L) was performed with immuno-turbidimetry assays (Randox Laboratories; 55 Diamond Road, Crumlin Co. Antrim, Northern Ireland, UK), and erythrocyte sedimentation rate (mm/h) was measured with sedisystem BD kit, following Wintrobe method [33].

Following the manufacturer’s instructions for commercial quantitative enzyme-linked immunoassay technique (ELISA) kits, we determined soluble levels of anti-cyclic citrullinated peptide antibodies (anti-CCP IU/mL, FCCP600 Axis-Shield Diagnostics Ltd, Dundee, Scotland) and basal insulin μIU/mL, (80-INSHU-E01.1. ALPCO 26-G Keewaydin Drive, Salem, NH 03079); the sensitivity of the assays are 1.04 IU/mL and 0.399 μIU/mL, respectively.

Quantitative determination of the ligands of chemokine receptors levels was also done with ELISA kits. For CCL2 pg/mL, (DCP00 R&D Systems Inc. 614 McKinley Place NE, Minneapolis, MN 55413, USA) and RvE1 μg/mL (MBS286046 My BioSource, Inc. P.O. Box 153308 San Diego, CA 92195–3308 USA), the sensitivity of the assays are 2.3 pg/mL and 0.1 μg/mL, respectively.

#### Subjects’ IR status

Indirect indexes for insulin sensitivity/resistance were estimated following these math equations [34]:

**Table:** 

Index	Equation
HOMA-IR	= [fasting serum glucose mg/dL×(fasting serum insulin μIU/mL)/405]
Quantitative insulin sensitivity check index, QUICKI	= [1/(log10(fasting serum insulin, μIU/mL) + log10(fasting glucose, mg/dL)]
β-cell function index, HOMA-B	= [(20×insulin μIU/mL)/(glucose mmol/L– 3.5)]
Basal disposition index DI	= [HOMA-B/HOMA-IR]

#### Chemokines system (CCR2/CCL2 and CMKLR1/RvE1) assessment

*Relative expression of CCR2 and CMKLR1 analysis*:

- Total *RNA* isolation: mononuclear cells from anticoagulated venous blood samples were isolated by density gradient using a separating solution (AXIS SHIELD PO Box 6863 Rodelokka, 0504 Oslo, Norway). Without delay total *RNA* was isolated using TRIzol® LS Reagent (Ambion RNA Life Technologies, 5791 Van Allen Way, Carlsbad, CA 92008) based on the single-step *RNA* isolation modified method reported by Chomczynski and Sacchi, 1987 [35].
- Total *RNA* obtained purity and concentration were measured with the Thermo Scientific™ Nanodrop™ ONE^C^ Spectrophotometer microvolume UV-Vis (Thermo Fisher Scientific, Waltman MA), and the Invitrogen Qubit 3 Fluorometer and Qubit® RNA HS Assay kit cat. Q32855 QIAGEN.
- Then, complementary *DNA* synthesis (*cDNA*) was performed with 100 ng of each total RNA sample using a reaction size of 10 *μ*L, with DEPC treated water, oligo (dT)12-18 primer (2.5 μM), dNTP mix (0.5 mM), RNaseOUT (1 U), DTT (2.5 mM), first standard buffer (1X), and 2.0 U of SuperScript® IV First-Strand Synthesis System kit (Invitrogen™. 5791 Van Allen Way, Carlsbad, California 92008 CAT, 8080093) and stored at −20°C until used for expression analyses.
- Real-Time Quantitative Polymerase Chain Reaction (RT-qPCR) measurements. Briefly explained, in one same experiment each *CCR2*, *CMKLR1* and *RPS28* genes relative expression was performed in a final reaction volume of 10 *μ*L (10 *μ*M forward and reverse primer, 500 nM ROX, 1X SYBR Green qPCR master mix, and 1000 ng of cDNA). The conditions of the reaction were as follows: holding 95°C/10 min, cycling (35 cycles of 95°C/15 s, 60°C/60 s), and melt curves 95°C/15 s, 60°C/60 s, and 95°C/15 s, was conducted using Rotor-Gene Q 5-Plex HRM detection system (QIAGEN).
- Expression of target genes *CCR2* and *CMKLR1* were normalized by the *RPS28* housekeeping gene, while sequence-specific forward and reverse primers were:

**Table:** 

Gen	Sequence-specific forward primer	Sequence-specific reverse primer
*CCR2* (NG_021428)	5’-GTGTGTGGAGGTCCAGGAGT-3’	5’-TTTCCTTTTCCACGACCATC-3’
*CMKLR1* (NM_001142343)	5’-GTGGTGGTCTACAGCATCGT-3’	5’-ATGGCGGCATAGGTGATATGG-3’
*RPS28* (NG_050637)	5’-GGTCTGTCACAGTCTGCTCC-3’	5’-CATCTCAGTTACGTGTGGCG-3’

To ensure data accuracy, experiments were done in duplicate, and blank and internal controls were included in all reactions. A threshold cycle (C_T_) value was determined from each amplification plot. Data was collected with applications of Rotor-Gene Q 5-Plex detector software (QIAGEN). The increase of relative expression in the target genes was calculated using the comparative C_T_ method with 2^−ΔΔC^_T_ equation [36].

### Statistical analysis

Data was analyzed with statistical software packages IBM SPSS Statistics v26 (IBM Inc., Chicago, IL, USA) and Graph-Pad Prism v6.01 (2014 Inc. 2236 Beach Avenue Jolla, CA 92037). The normal distribution of adiposity status and metabolic variables was evaluated and confirmed with *Z* Kolmogorov-Smirnov test. Results were given as median, minimum and maximum, percentages, mean ± SD or standard error of the mean (SEM). The subjects’ clinical assessments, status of body adiposity IR indexes, immuno-metabolic and chemokine profiles of the study groups were compared with Kruskal-Wallis *H* test or one-way ANOVA with HSD Tukey post hoc, Student *t* test or Mann-Whitney *U* test and Spearman *rho* correlation tests. Levels of relative expression between study-groups were compared with a Kruskal-Wallis *H* test. Additionally, Pearson χ^2^ test was performed for comparisons between observed frequencies in study groups. A two-tailed *P* value < 0.05 was considered statistically significant.

## Results and discussion

### Clinical assessments of RA patients

We studied 138 control subjects and 138 patients with RA groups, then subjects were classified according to the presence of IR. RA subjects had an increased frequency of IR compared to controls, 42% (58/138) *versus*. 21% (29/138) *P* < 0.001. As expected, we observed a significant association between BMI and IR in the control group Table 1. Since, 48% and 52% of subjects, with pre-obesity and obesity were classified with IR, while none of the control subjects with normal weight had IR (*P* = 0.0002). In contrast, 23% (13/58) of RA patients in the normal weight category had IR, and association between the WHO BMI categories and IR was not significant in the RA group (*P* = 0.328).

**Table 1:** Clinical assessments of the study groups.

Measurement	Control group		Rheumatoid arthritis group		*P value*
	Without IR	With IR ^a^	Without IR	With IR ^a^	
**n (%)**	109 (79)	**29 (21)**	80 (58)	**58 (42)**	**< 0.001**^**c**^
**Age (years)** ^**b**^	**43 [18 – 61]**	44 [19 – 58]	**45 [18 – 63]**	**49 [22 – 63]**	**0.020** ^**f**^
**Smoking history of tobacco n (%)** ^**c**^	—	—	6 (8)	2 (4)	0.052
***World Health Organization classification criteria*** ^***c***^					
Normal weight [BMI 18.5–24.9 kg/m^2^] n(%)	**40 (37)**	**0 (0)**	**27 (34)**	**13 (23)**	**0.003**
Pre-obesity [BMI 25.0–29.9 kg/m^2^] n (%)	**44 (40)**	**14 (48)**	**31 (39)**	**28 (48)**	
Obesity [BMI ≥ 30.0 kg/m^2^] n (%)	**25 (23)**	**15 (52)**	**22 (27)**	**17 (29)**	
***Serum and inflammation markers levels*** ^***b***^					
Albumin (g/dL)	4.8 ± 0.64	**4.5 ± 0.40**	4.8 ± 0.65	**5.0 ± 0.66**	**0.013**
Aspartate aminotransferase (U/L)	**18.7 ± 8.1**	**24.8 ± 9.6**	21.4 ± 9.0	**25.5 ± 11.5**	**< 0.001** ^**e**^
C-reactive protein (mg/L)	**6.2 ± 3.1**	**8.3 ± 4.3**	9.3 ± 12.0	**9.8 ± 6.9**	**< 0.001** ^**e**^
Erythrocyte sedimentation rate (mm/h)	**13.3 ± 8.5**	15.4 ± 8.4	**22.3 ± 13.8**	**26.1 ± 14.8**	**< 0.001** ^**f**^
TNFα (pg/mL)	25.9 ± 17.2	65.7 ± 99.1	28.9 ± 18.6	27.1 ± 25.4	0.122
Rheumatoid factor (IU/mL) ^d^	—	—	104 ± 154	195 ± 261	0.057
Anti-CCP (U/mL) ^d^	—	—	52 ± 92	74 ± 129	0.407

Control group n = 138, RA group n = 138. The results are shown in $\bar{x}$ ± SD, percentages (%) or median [min-max].

^a^ Classified based on Stern criteria. *P* values were calculated using

^b^ Kruskal-Wallis *H* test,

^c^ Pearson χ^2^ test,

^d ^Mann-Whitney *U* test (*P* < 0.05 was significant). Bold numbers show differences between groups:

^e^ control group without IR *versus* both groups with IR.

^f^ Control group without IR *versus* both RA groups. Abbreviations: IR: insulin resistance; BMI: body mass index; TNFα: tumor necrosis factor alpha; Anti-CCP: anti-cyclic citrulinate peptide antibodies. None of RA patients are current active or passive smokers.

Next, we analyzed the association between RA clinical characteristics, disease activity and inflammation markers with IR Tables 1 and 2. The inflammation markers levels were higher in both RA and control with IR compared to the control group without IR Table 1. There was no difference in the disease activity score, distribution of disease activity groups and treatment schemes between RA with and without IR Table 2. Also, there was no association between disease activity and obesity status in the RA groups S1 Table. Interestingly, anti-CCP were more frequent in subjects with RA and IR compared to RA without IR, 76% (44/58) *versus* 59% (47/80) *P* = 0.036, respectively.

**Table 2:** DMARDs treatments and disease activity of the rheumatoid arthritis patients.

**Study Group**	**Rheumatoid arthritis groups**								***P value***
	**Without IR**				**With IR** ^**a**^				
**N**	80				58				—
**Disease duration (years)**	6 [2–39]				5 [1–34]				0.394^d^
**Patients {DMARDs dose mg/week}**									
*Monotherapy*									
Methotrexate	21% {2.5–20}				21% {12.5–20}				**—**
Sulfasalazine	3% {14000}				5% {14000}				**—**
*Double therapy*									
Methotrexate + Chloroquine	15% {7.5–20 + 1050}				21% {2.5–15 +1050}				**—**
Methotrexate + Sulfasalazine	25% {2.5–17.5 + 3500–21000}				32% {2.5–30 + 500–21000}				**—**
Chloroquine + Sulfasalazine	3% {1050 + 10500}				0%				**—**
*Triple therapy*									
Methotrexate + Chloroquine + Sulfasalazine	33% {5–25 + 600–1050 + 2000–14000}				21% {15–25 + 1050 + 10500–21000}				**—**
**DAS28-CRP score**	2.81 [1.28–6.2]				2.70 [1.21–6.04]				0.238^d^
**Disease activity categories**	**Remission**	**Low**	**Moderate**	**High**	**Remission**	**Low**	**Moderate**	**High**	
*DAS28-CRP score cut-off*	*≤ 2*.*6*	*2*.*7–3*.*2*	*3*.*2–5*.*1*	*> 5*.*1*	*≤ 2*.*6*	*2*.*7–3*.*2*	*3*.*2–5*.*1*	*> 5*.*1*	
n (%)	16 (20)	30 (37)	24 (30)	10 (13)	27 (46)	16 (27)	11 (19)	4 (8)	0.312^c^
***Serum markers***	Positive		Negative		Positive		Negative		
Anti-CCP n (%)	**47 (59)**		**33 (41)**		**44 (76)**		**14 (24)**		**0.036**^c^
Rheumatoid factor n (%)	46 (58)		34 (42)		38 (65)		20 (35)		0.494^c^

RA group n = 138. The results are shown in % {min–max}, n (%) or median [min-max].

^a^ Classified based on Stern criteria. *P* values were calculated using

^c^ Pearson χ^2^ test,

^d^ Mann-Whitney *U* test (*P* < 0.05 was significant). Abbreviations: Anti-CCP: anti-cyclic citrulinate peptide antibodies; DAS28-CRP: disease activity score on 28 joints with C-reactive protein. DMARDs: Disease-modifying anti-rheumatic drugs therapy; IR: insulin resistance.

Here, we confirmed the strong association between RA and IR. While the link between IR and RA is not fully understood, it is clear that the pathogenic events that lead to IR are amplified by the inflammatory response in RA. The fact that 23% of normal weight RA subjects have IR, supports the idea that the development of IR in RA, it is not completely dependent on fat mass acumulation, and more likely driven by a subclinical chronical inflammatory process. This is further supporter by lack of association between RA disease actvity and IR, and the increased inflammation markers RA with IR. Moreover, there was no difference in the treatment schemes used in both group of patients, indicating that the severity and disease control was similar between both RA groups.

Interestingly, similar to other studies, we observed an association between ACPA positivity and insulin resistance [6, 37–39]. While, currently there is no data to support that adipose tissue as a target of ACPA, this autoantibody system supports several mechanism important for the development of IR. ACPA are able induce the production of pro-inflammatory citokines by PBMC and macrophages (i.e.TNFα, IL-6, IL-12 and IL-17) [40]. On the other hand, ACPA positive RA have higher numbers of infiltrating adipose tissue macrophages [41].

In RA’s pathogenesis, several processes are involved, as dysregulation of the metabolic process occurs at different stages of the disease. Supported by previous studies, we know a high BMI is linked to increased disease activity and disability at disease onset, being an independent factor of less responsive to therapy [42, 43]. However, not all studies led to a common conclusion, and some of them gave inconsistent results [44].

#### Insulin resistance favors increased body adiposity status

Body adiposity *status* of RA patients with or without IR was different from control groups. This difference was more evident in the body dimensions and total and trunk storage fat mass between the control group without IR and the RA group with IR Table 3.

**Table 3:** Adiposity status in study subjects.

Measurement	Control group		Rheumatoid arthritis group		*P value*
	Without IR	With IR ^a^	Without IR	With IR ^a^	
**n**	109	29	80	58	—
**BMI (kg/m**^**2**^**)** ^*b*^^	**27.1 [18.5–43.7]**	**31.6 [25.7–41.8]**	**27.1 [18.5–39.0]**	28.8 [18.5–43.3]	**<0.001**^**h**^
***Storage of body fat mass*** ^*b^*^					
Body weight (kg)	**69.4 ± 13.3**	**80.7 ± 13.2**	**66.8 ± 11.9**	**70.6 ± 13.2**	**<0.004** ^**g**^
Total body fat mass (%)	**34.9 ± 7.4**	**39.3 ± 4.8**	**35.2 ± 6.9**	37.6 ± 7.4	**<0.036** ^**h**^
Total body fat mass (kg)	**25.2 ± 9.8**	**32.2 ± 9.1**	**25.3 ± 9.3**	27.4 ± 10.0	**<0.006** ^**h**^
Total body non-fat mass (%)	65.0 ± 7.4	**60.8** ± **4.8**	**64.7** ± **6.9**	62.3 ± 7.5	**0.006** ^**i**^
Total body non-fat mass (kg)	47.9 ± 3.8	48.6 ± 4.8	42.6 ± 4.6	43.2 ± 4.1	0.377
***Distribution of body fat mass*** ^***b***^					
Trunk fat mass (%)	**47.3 ± 5.7**	**46.9 ± 4.6**	**33.3 ± 17.2**	**35.1 ± 23.0**	**0.001** ^**g**^
Trunk fat mass (kg)	**12.2 ± 5.2**	**15.5 ± 4.8**	**11.6 ± 4.8**	**15.0 ± 14.0**	**0.018** ^**g**^
Trunk non-fat mass (%)	55.6 ± 7.0	56.4 ± 1.3	56.1 ± 5.8	55.7 ± 6.9	0.952
Trunk non-fat mass (kg)	**24.9 ± 3.2**	**27.3 ± 2.3**	**23.8 ± 3.4**	**24.0 ± 3.5**	**0.000** ^**g**^
Upper limbs fat mass (%)	**10.3 ± 1.6**	**11.6 ± 1.7**	**10.3 ± 1.8**	10.7 ± 1.3	**0.002** ^**h**^
Upper limbs fat mass (kg)	**2.7 ± 1.4**	**3.8 ± 1.5**	**2.6 ± 1.1**	3.0 ± 1.4	**<0.001** ^**h**^
Lower limbs fat mass (%)	42.0 ± 5.9	39.7 ± 4.4	41.5 ± 8.4	44.9 25.9	0.469
Lower limbs fat mass (kg)	10.2 ± 3.2	**12.6 ±2.9**	**9.9 ± 2.6**	**11.6 ± 5.3**	**0.001** ^**j**^
*Skinfold thickness (mm)* ^*b^*^					
Abdominal	23.1 ± 9.8	26.6 ± 9.6	26. ± 10.1	27.1 ±10.2	0.066
Bicipital	**13.0 ± 6.8**	16.1 ± 4.6	15.4 ± 7.1	**17.1 ± 8.1**	**0.002** ^**k**^
Tricipital	**22.5 ± 6.9**	25.6 ± 6.6	24.7 ± 8.2	**26.6 ± 8.0**	**0.006** ^**k**^
Subscapular	**21.2 ± 8.68**	24.5 ± 7.58	24.4 ± 9.20	**26.4 ± 9.40**	**0.002** ^**k**^
Suprailiac ^*b*^	**19.8 ± 8.4**	**26.3 ± 9.7**	22.3 ± 9.8	**26.3 ± 9.5**	**<0.005** ^**e**^
***Body dimensions (cm)*** ^*b^*^					
Waist circumference	**88.8 ± 14.1**	**96.8 ± 12.8**	89.2 ± 11.5	**95.0 ± 13.5**	**<0.022** ^**e**^
Hip circumference	**105.7 ± 9.9**	**113.2 ± 10.8**	**104.2 ± 9.8**	**106.7 ± 10.3**	**<0.026** ^**g**^
Sagittal abdominal diameter	**19.9 ± 3.8**	**22.2 ± 3.8**	20.9 ± 2.7	**22.2 ± 3.6**	**<0.013** ^**e**^
Coronal abdominal diameter^*b*^	**29.9 ± 5.6**	**32.9 ± 5.9**	30.7 ± 4.2	**32.5 ± 6.6**	**<0.047** ^**e**^
Visceral area (cm^2^) ^*b*^	647 ± 83	819 ± 176	849 ± 95	805 ± 125	0.439

Control group n = 138, RA group n = 138. The results are shown in $\bar{x}$ ± SD, percentages (%) or median [min-max].

^a^ Classified based on Stern criteria. *P* values were calculated using

^b^ Kruskal-Wallis *H* test or

^b^^ANOVA, HSD Tukey post-hoc (*P* < 0.05 was significant). Abbreviations: IR: insulin resistance; BMI: body mass index. Bold numbers show differences between groups:

^e^ Control group without IR *versus* both groups with IR.

^g ^Control groups *versus* both RA groups.

^h^ Control group without IR *versus* control group with IR and RA group without IR.

^i^ Control group with IR *versus* RA without IR group.

^j^ Control group with IR *versus* both RA groups.

^k^ Control group without IR versus RA group with IR.

**Fig 1:**
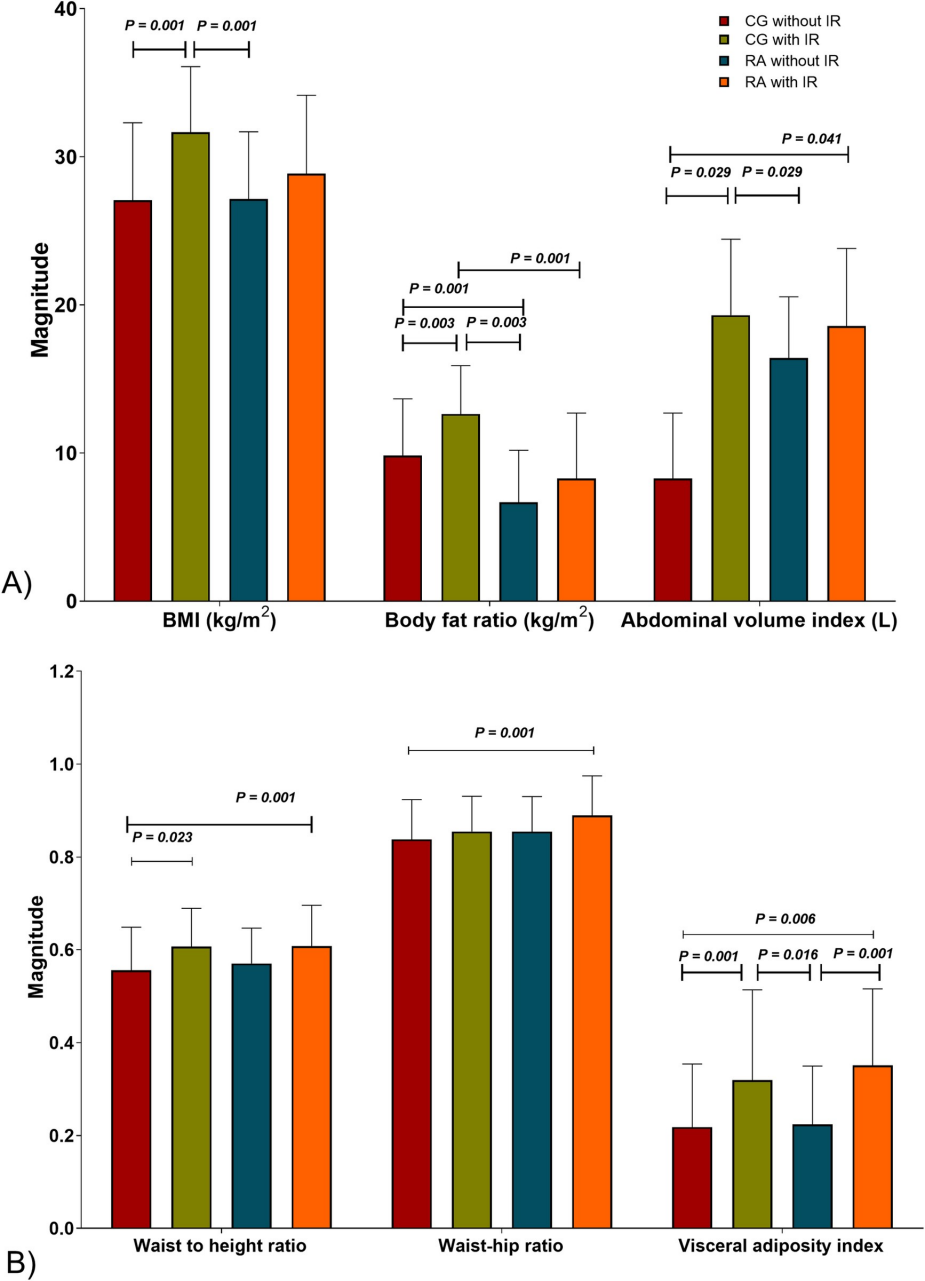
Obesity indexes in study groups. A) Body dimension ratios. B) Storage of body fat mass. CG without IR n = 109, CG with IR n = 29, RA without IR n = 80, RA with IR n = 58. IR is classified based on Stern criteria. The results are shown in $\bar{\text{x}}$ ± SD. *P* values were calculated using ANOVA, HSD Tukey post-hoc, (*P* < 0.05 was significant). Abbreviations: CG: control group; RA: rheumatoid arthritis; IR: insulin resistance; BMI: body mass index.

Interestingly, we observed higher magnitudes of body and abdominal adiposity indexes in the RA group with IR *versus* the control group without IR. Concerning the adiposity indexes that evaluate the abdominal distribution of fat mass, both groups with IR had the highest values; however, the differences were more noticeable between the control group with IR *versus* those without IR Fig 1.

Most RA patients develop metabolic comorbidities, during its clinical progression has been associated with IR [6–8] and alterations about the body pathologic redistribution of fat mass [9, 10]. Extensive studies have been carried out to investigate the association between obesity and RA’s risk; nevertheless, the current reported original studies’ results remain diverging [44].

In this context, IR is a common feature throughout obesity development, but the definition and classification of obesity are still debatable. The WHO consistently classified individuals into four categories based on their BMI (kg/m^2^): underweight BMI < 18.50, normal weight BMI = 18.50–24.99, pre-obesity = BMI 25.00–29.99, and obesity BMI ≥30.00. However, this single parameter often underestimates the risk of metabolic disease. This study observed that some individuals classified as pre-obesity or with obesity do not display an increased cardiovascular risk nor any metabolic alteration.

For this concern, it is essential to consider where fat is stored: in intra-abdominal (visceral) or subcutaneous (fat stored beneath the skin) regions. Previous studies reported that these different adipose tissue depots showed metabolic implications and effects [45]. The former has been more commonly linked to hypertension and T2D, while the latter conferred a neutral or even protective effect against metabolic disease [44].

Evidence has shown the pleiotropic nature of fat depots [46]. Moreover, studies on animal models have shown remarkable results. About this topic, Thien T. Tran, 2008, *et al*., reported that intra-abdominal WAT transplantation to the subcutaneous region improves metabolism by reducing body weight and overall fat mass, also increasing insulin sensitivity and body glucose uptake [47].

On the other hand, in our study, the BMI and IR prevalence (42%) of RA patients were similar as previous reports [8, 42, 43] Also, RA patients with IR included individuals classified with normal weight, pre-obesity, and obesity; in contrast, no one in the control group with IR was classified with normal weight. Moreover, we have shown a detailed measurement of fat mass quantity and its distribution. It is essential to highlight that RA patients with IR showed the largest central body dimensions, obesity indexes, and dyslipidemic profile Table 3 and S1 Fig.

Even though the common conception is that greater intra-abdominal fat mass is the key to developing metabolic dysregulation. The RA patients with IR in our study group show that metabolic alterations can develop independently of the fat mass quantity and location. Our results could be explain with the evidence by Foster & Pagliasotti, 2012, and others, who concluded that adipose tissue intrinsic properties and function, regardless of mass and location, are responsible for metabolic dysregulation [45–48]. Based on this, we suggest that exists an intrinsic relation between RA and RI regardless of adiposity.

#### Insulin sensitivity status in IR groups: Aside from HOMA-IR, complementary indexes depicted adequately

**Fig 2:**
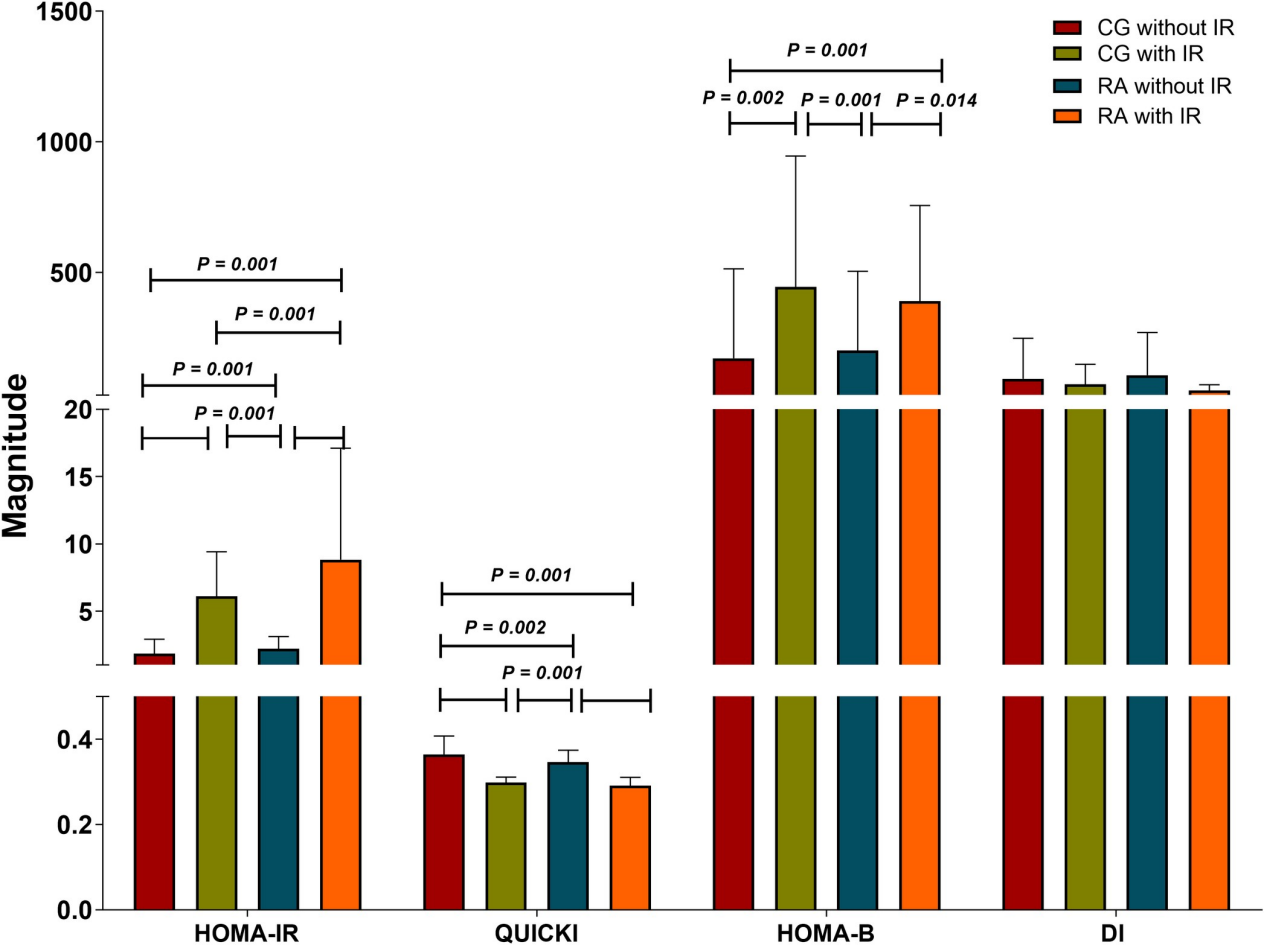
Insulin sensitivity *status* in the study groups. CG without IR n = 109, CG with IR n = 29, RA without IR n = 80, RA with IR n = 58. IR classified based on Stern criteria. The results are show in $\bar{\text{x}}$ ± SD. *P* values were calculated using Kruskal-Wallis *H* test, (*P <* 0.05 was significant). Abbreviations: CG: control group; RA: rheumatoid arthritis; IR: insulin resistance; HOMA-IR: homeostasis model assessment of insulin resistance; QUICKI: quantitative insulin sensitivity check index; HOMA-B: homeostatic model assessment of β-cell; DI: basal disposition index.

In addition to HOMA-IR, to highlight insulin sensitivity status QUICKI, HOMA-B and DI indexes were considered. Although we found that QUICKI and HOMA-B indexes mirrored the results obtained with HOMA-IR in all groups, the latter was able to more accurately discriminate the differences in all groups, especially between the IR groups. Meanwhile, DI values were similar in all groups Fig 2.

In the RA with IR group, the correlations were evident on HOMA-IR and QUICKI indexes not only with storage and distribution of the body fat mass, but also with body dimensions and obesity indexes. CRP and lipid profile values correlated as well with the insulin sensitivity indexes S2 Table.

Although conceptually different, HOMA-IR and QUICKI indexes are mathematically proportionally related (QUICKI = 1/(log [insulinemia (μU/mL)] + log [glycemia (mmol/L)]). Since the design of QUICKI by Arie Katz, *et*. *al*., 2000, it has been recognized as a reliable, reproducible, and accurate index of insulin sensitivity with a strong positive predictive value in healthy insulin-sensitive individuals. It has a high correlation with the gold standard of IR evaluation (hyperinsulinemic-euglycemic clamp) [49].

Along with those mentioned above, we identified in the RA group with IR an accumulative effect of the inflammatory and lipid profiles and the magnitude of IR indexes. These measurements compare with other studies that contain evidence of insulin sensitivity/resistance assessment over a wide range of assorted populations [34, 50–53]. At this point, we could suggest that when IR has been established in RA patients, they show evidence of an unfavorable metabolic and inflammatory state probably potentiated by the pathological expansion and distribution of body adipose tissue.

#### The relative expression levels of *CCR2* and *CMKLR1* matched the serum levels of its chemokine ligands CCL2 and RvE1

**Fig 3:**
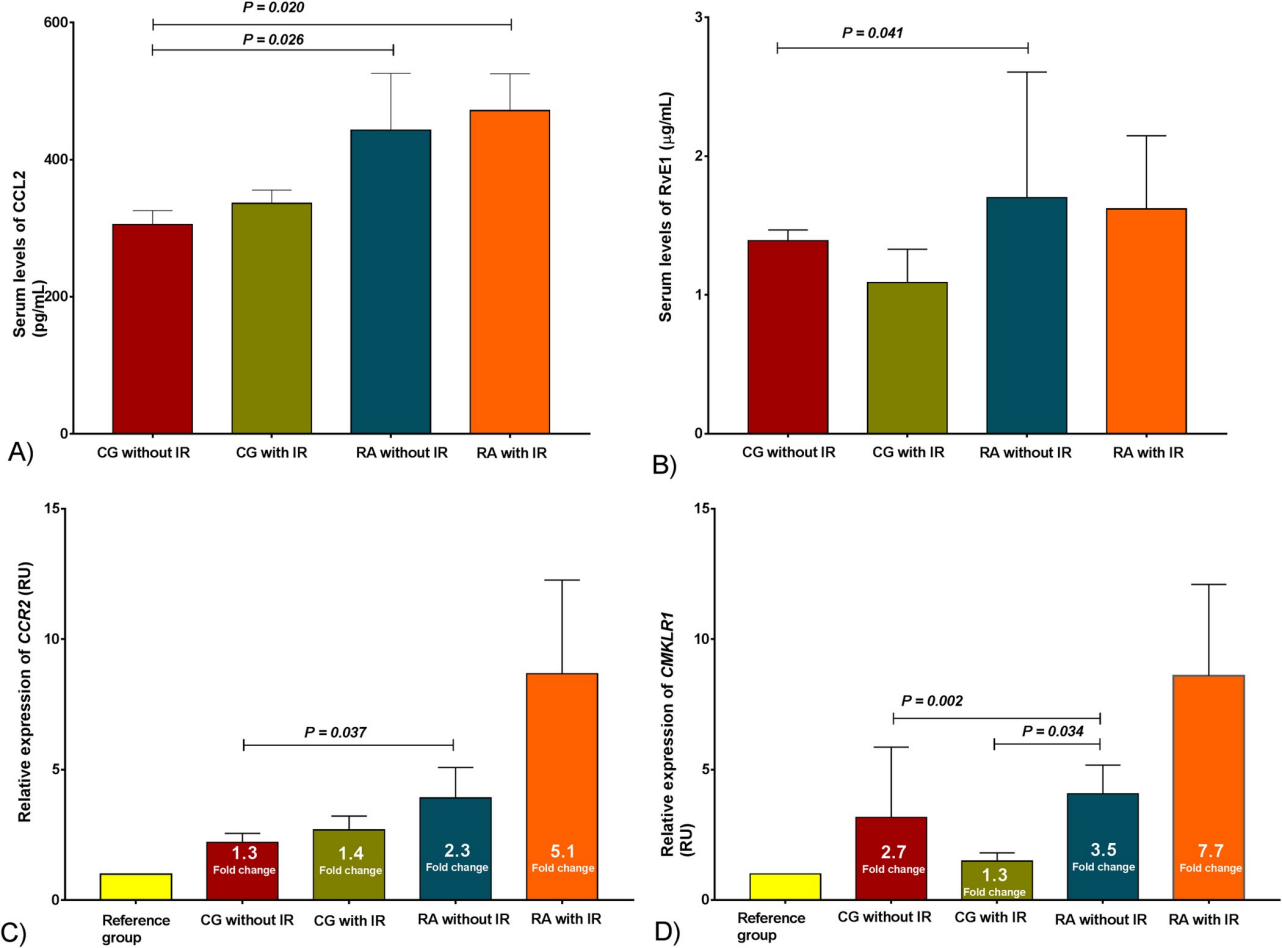
Chemokines system (*CCR2*/CCL2, *CMKLR1*/RvE1) assessment in the study group. A) Serum levels of CCL2, B) Serum levels of RvE1, the results are show in $\bar{\text{x}}$ ± SD. C) Relative expression of *CCR2* and D) Relative expression of *CMKLR1*, the results are show in $\bar{\text{x}}$ ± SEM. *P* values were calculated using Kruskal-Wallis *H* test, (P < 0.05 was significant). CG without IR n = 109, CG with IR n = 29, RA without IR n = 80, RA with IR n = 58. IR classified based on Stern criteria. Abbreviations: CG: control group; RA: rheumatoid arthritis; IR: insulin resistance; *CCR2*: C-C chemokine receptor type 2; *CMKLR1*: Chemokine like receptor 1; CCL2: C-C motif chemokine ligand 2; RvE1: Resolvin E1; SEM: standard error of the mean.

The serum levels of CCL2 were higher in both RA groups *versus* control group without IR (443 ± 83 and 336 ± 19 pg/mL, *versus* 305 ± 20 pg/mL respectively, Fig 3A). Interestingly, RvE1 serum levels increased in RA group without IR *versus* control group without IR (1.70 ± 0.17 *versus* 1.39 ± 0.07 μg/mL, respectively) Fig 3B.

Although RA patients showed 2.3- and 5.1-fold change in the relative expression of *CCR2*, only in the RA group with IR was the expression higher than the control group without IR Fig 3C. On the other hand, *CMKLR1* relative expression showed differences between the RA group without IR and control groups. Also, we observed a 1.3- and 2.7-fold change in control groups with and without IR and a 3.5-fold change in the RA without IR group Fig 3D.

#### Chemokine receptors relative expression were associated with immuno-metabolic markers

In particular, *CCR2* and *CMKLR1* relative expressions correlate between them. We also found that *CCR2* relative expression correlates negatively with apolipoprotein levels and with abdominal fat mass storage, whereas *CMKLR1* relative expression correlates positively with some components of the lipid profile and with total body non-fat mass, specially the one stored in the trunk. Other relevant correlations with insulin sensitivity indexes and inflammatory markers are shown in Table 4.

**Table 4:** *CCR2* relative expression correlations in study group.

**Measurements**	***CCR2 relative expression* (2**^**−ΔC**^_**T**_ **method)**	
	***Rho***	***P***
***CMKLR1* relative expression**	0.331	0.000
**HOMA-B**	0.548	0.019
**DI index**	0.497	0.036
**Apolipoprotein A-1 (mg/dL)**	− 0.217	0.014
**Apolipoprotein B (mg/dL)**	− 0.181	0.041
**Visceral area (cm**^**2**^**)**	− 0.273	0.048
**Coronal abdominal diameter (cm)**	− 0.178	0.044
**Measurements**	***CMKLR1 relative expression* (2**^**−ΔC**^_**T**_ **method)**	
	***Rho***	***P***
**CCL2 (pg/mL)**	0.224	0.042
**TNFα (pg/mL)**	0.418	0.048
**LDLc (mg/dL)**	0.168	0.045
**LDLc/HDLc**	0.171	0.042
**Total body non-fat mass (kg)**	0.173	0.039
**Trunk non-fat mass (%)**	0.189	0.024

Study group n = 276. *P* values were calculated using *rho* Spearman correlation test, (*P* < 0.05 was significant). Abbreviations: CCR2: C-C motif chemokine receptor 2; *CMKLR1*: chemerin-chemokine like receptor 1; HOMA-B: homeostatic model assessment of β-cell index; DI: basal disposition index; CCL2: C-C motif chemokine ligand 2; TNFα: tumor necrosis factor-alpha; HDLc, and LDLc: high and low-density lipoproteins cholesterol, respectively.

Regarding the chemokines, we observed increased levels of CCL2 in RA groups Fig 3A, as reported by Lin Zhang, 2015 in RA patients [54]. However, these levels did not correlate with body adiposity *status* measurements as observed in previous studies in subjects with obesity and IR [55].

Although RvE1 is an anti-inflammatory molecule, we found increased serum levels in the RA group without IR as showed previously [56]. Its levels were higher in individuals with obesity and IR than those without IR. As far as we know, CCL2 and RvE1 levels correlations have not been reported yet; therefore, there is no precedent for comparison.

The CCL2 levels found can be explained by an altered production of soluble mediators of chemokines systems, mainly secreted by the immune cells of adipose tissue with the following activation of several pro-inflammatory signaling pathways that interfere with insulin signaling, representing a crosstalk between immune cells and adipocytes [57, 58].

Previous studies have reported activation and resolution of inflammatory processes [59, 60]. In our research, at the conjunction of RA with IR and adiposopathy, we observed that serum levels of CCL2 and RvE1 had similar behavior, although having antagonistic effects. This convergence may exacerbate the chronic inflammation state, demonstrated by high CCL2 levels and compensatory RvE1 levels that cannot achieve inflammation resolution.

The relative expression pattern of *CCR2* and *CMKLR1* showed a parallel increase with their respective ligands’ levels (CCL2 and RvE1). RA patients presented higher relative expression compared to control individuals on both receptors Fig 3, which were associated with IR indexes Table 4. A suggested explanation for these results is that, as WAT expands, the CCR2/CCL2 chemokine system is activated and promotes transmigration of circulating monocytes into this tissue, which is polarized into pro-inflammatory M1 macrophages [61].

CMKLR1, as a chemoattractant receptor for the lipid mediator RvE1, plays a role in the migration of monocytes to sites of inflammation, for its expression is upregulated during the differentiation of monocytes into M1 macrophages. In contrast, it is undetectable in activated M2 macrophages [15, 62]. Nevertheless, not enough evidence exists about its regulation during different stages of inflammation or monocyte/macrophage activation in WAT.

The CCR2/CCL2 system has been studied because it participates on the recruitment of monocytes into WAT, with subsequent polarization into proinflammatory M1 macrophages [63]. Evidence-based on animal models demonstrate the central role of WAT in the preservation of the inflammatory response in the adiposopathy state, and the link between chemokines system axis and the development of IR [58, 64]; for example, in 2016 Kawano Y. reported that murine Ccr2 knockout models (M-Ccr2KO and Vil-Ccl2KO) with high fat diet (HFD)-induced IR, showed improved metabolic and inflammatory responses than wild types [9]. In perspective, according to our results and reported evidence, we agree that the hallmark of adiposopathy and IR is a constant chronic low-grade inflammatory process, in which circulating monocytes infiltrate WAT and polarize towards M1 macrophages, becoming the source of chemokines and their receptors, thus establishing a redundant inflammatory process that potentiates RA scenario.

Finally, the main contribution of this work is that the shown increase of *CCR2/*CCL2 and *CMKLR1*/RvE1 systems’ activity could consider them as potential indicators of inflammation severity in comorbidities like IR in RA patients. We suggest that in RA with IR, an increase in the chemokines systems levels signals an accumulative effect in the inflammatory process. Also, both CCL2 and RvE1 levels had a parallel response, analogous to the relative expression pattern of their receptors (*CCR2* and *CMKLR1*), which represent activation and resolution attempt of inflammation, respectively.

## Conclusions

We conclude that in RA with IR, the chemokine receptors expression pattern showed a parallel increase with their respective ligands. RA and IR in conjunction with the pathological quantity and distribution of body fat mass might exacerbate chronic inflammation. These results suggest that high CCL2 levels and compensatory RvE1 levels might not be enough to achieve inflammation resolution.

## Supporting information

S1 Table Disease activity and its relationship with treatment, insulin resistance and BMI. (DOCX)

S2 Table Insulin resistance *status* correlations in RA with IR study group. (DOCX)

S1 FigLipid profile in the study groups.A) Lipids profile, B) Metabolic markers, C) Cholesterol ratios. The results are show in $\bar{\text{x}}$ ± SD. Kruskal-Wallis *H* test, (P < 0.05 was significant). Bold lines (**—**) show differences between groups. Abbreviations: RA: rheumatoid arthritis; IR: insulin resistance; HDLc, LDLc and VLDLc (high, low and very low-density lipoproteins cholesterol, respectively); Apo: apolipoprotein.(TIF)

S1 Data (TIF)

S2 Data IR *status* correlations with adiposity in study group. (DOCX)

S3 Data IR status correlations with immuno-metabolic markers in study group. (DOCX)
