# Supplementary material for: CCR2/CCL2 and CMKLR1/RvE1 chemokines system levels are associated with insulin resistance in rheumatoid arthritis
Source: PLoS One. 2021 Jan 28;16(1):e0246054. doi: 10.1371/journal.pone.0246054 (PMC7842933; doi:10.1371/journal.pone.0246054)
Supplement: S1 Table — (DOCX) [file pone.0246054.s001.docx]

**S1 Table. Disease activity and its relationship with treatment, insulin resistance and BMI.**

| Measurement | RA disease activity categories  (DAS28-CRP score cut-off) | | | | *P value* |
| --- | --- | --- | --- | --- | --- |
|  | **Remission**  ≤ 2.6 | **Low**  2.7 – 3.2 | **Moderate**  3.2 – 5.1 | **High**  > 5.1 |  |
| ***RA DMARDs therapy*** | | | | |  |
| Monotherapy (n = 34) | 53.8 % | 7.7 % | 38.5 % | 0 % | 0.358 ^c^ |
| Double therapy (n = 65) | 34.8 % | 30.4 % | 26.1 % | 8.7 % |  |
| Triple therapy (n = 39) | 61.5 % | 7.7 % | 23.1 % | 7.7 % |  |
| ***Serum marker*** |  |  |  |  | 0.296 ^c^ |
| ACPA negative (n = 47) | 50.0 | 6.3 | 31.3 | 12.5 |  |
| ACPA positive (n = 91) | 45.2 | 25.8 | 25.8 | 3.2 |  |
| ***RA groups*** | | | | |  |
| RA without IR ^a^ (n = 80) | 43.5 % | 13.0 % | 30.4 % | 13.0 % | 0.312 ^c^ |
| RA with IR ^a^ (n = 58) | 46.2 % | 26.9 % | 23.1 % | 3.8 % |  |
| ***World Health Organization*** ***classification criteria [BMI cut-off]*** | | | | |  |
| Normal weight [18.50 – 24.99 kg/m^2^] (n = 40) | 57.1 % | 14.3 % | 19.0 % | 9.5% | 0.807 ^c^ |
| Pre-obesity [25.00 – 29.99 kg/m^2^] (n = 59) | 37.9 % | 20.7 % | 34.5% | 6.9% |  |
| Obesity [ > 30.00 kg/m^2^] (n = 39) | 40.9 % | 18.2 % | 27.3% | 13.6% |  |

RA group n = 138. The results are shown in percentages (%). ^a^ Classified based on Stern criteria. ^c^ P values were calculated using Pearson χ^2^ test (P < 0.05 was significant). Abbreviations. DAS28-CRP: disease activity score on 28 joints with C-reactive protein; DMARDs: Disease-modifying anti-rheumatic drugs therapy; ACPA: anti-cyclic citrulinate peptide antibodies; RA: rheumatoid arthritis; IR: insulin resistance; BMI: body mass index.
