# Supplementary material for: CCR2/CCL2 and CMKLR1/RvE1 chemokines system levels are associated with insulin resistance in rheumatoid arthritis
Source: PLoS One. 2021 Jan 28;16(1):e0246054. doi: 10.1371/journal.pone.0246054 (PMC7842933; doi:10.1371/journal.pone.0246054)
Supplement: S2 Table — (DOCX) [file pone.0246054.s002.docx]

S2 Table. Insulin resistance *status* correlations in RA with IR study group.

| Measurements | HOMA-IR | | QUICKI | | HOMA-B | | DI | |
| --- | --- | --- | --- | --- | --- | --- | --- | --- |
|  | *rho* | *P* | *rho* | *P* | *rho* | *P* | *rho* | *P* |
| ***Storage of body fat mass*** | | | | | | | | |
| Body weight (kg) | 0.176 | **0.003** | − 0.182 | **0.003** | − | − | − | − |
| Total body fat mass (%) | 0.164 | **0.006** | − 0.170 | **0.005** | − | − | − 0.127 | **0.036** |
| Total body fat mass (kg) | 0.150 | **0.013** | − 0.153 | **0.010** | − | − | − | − |
| ***Distribution of body fat mass*** | | | | | | | | |
| Trunk fat mass (kg) | 0.154 | **0.011** | − 0.160 | **0.008** | − | − | − | − |
| Trunk fat mass (%) | − 0.182 | **0.003** | 0.178 | **0.003** | − 0.183 | **0.003** | − | − |
| Upper limbs fat mass (%) | 0.269 | **0.001** | − 0.271 | **0.001** | 0.160 | **0.008** | − | − |
| Upper limbs fat mass (kg) | 0.207 | **0.001** | − 0.213 | **0.001** | − | − | − 0.122 | **0.045** |
| Lower limbs fat mass (kg) | 0.222 | **0.001** | − 0.228 | **0.001** | − | − | − 0.146 | **0.017** |
| ***Body dimensions (cm)*** | | | | | | | | |
| Waist circumference (cm) | 0.245 | **0.001** | − 0.249 | **0.001** | − | − | − 0.251 | **0.001** |
| Hip circumference (cm) | 0.146 | **0.001** | − 0.152 | **0.012** | − | − | − | − |
| Coronal diameter (cm) | 0.159 | **0.008** | −0.162 | **0.007** | − | − | − 0.187 | **0.002** |
| ***Obesity indexes*** | | | | | | | | |
| BMI (kg/m^2^) | 0.252 | **0.001** | − 0.258 | **0.001** | − | − | − 0.127 | **0.037** |
| Body fat ratio | 0.118 | **0.049** | − 0.123 | **0.042** | − | − | − 0.157 | **0.010** |
| Waist to height ratio | 0.272 | **0.001** | − 0.275 | **0.001** | − | − | − 0.261 | **0.001** |
| Waist-hip ratio | 0.203 | **0.001** | − 0.201 | **0.001** | − | − | − 0.288 | **0.001** |
| Visceral area (cm^2^) | − | − | − | − | − | − | 0.140 | **0.033** |
| Abdominal volume index | 0.242 | **0.001** | − 0.246 | **0.001** | − | − | − 0.244 | **0.001** |
| ***Lipid profile*** (mg/dL) |  |  |  |  |  |  |  |  |
| Triglycerides | 0.277 | **0.001** | − 0.276 | **0.003** | − | − | − 0.201 | **0.001** |
| VLDLc | 0.277 | **0.001** | − 0.276 | **0.001** | − | − | − 0.201 | **0.001** |
| Triglycerides/HDL | 0.385 | **0.001** | − 0.382 | **0.001** | 0.189 | **0.002** | − 0.197 | **0.001** |
| HDLc | − 0.234 | **0.001** | − 0.231 | **0.001** | − 0.199 | **0.001** | − | − |
| Apo A-1 | − 0.135 | **0.029** | 0.135 | **0.029** | − 0.179 | **0.004** | − | − |
| LDLc/HDLc | 0.134 | **0.026** | − 0.130 | **0.031** | − | − | − | − |
| ***Inflammation markers*** |  |  |  |  |  |  |  |  |
| CRP (mg/L) | 0.198 | **0.001** | − 0.195 | **0.012** | − | − | − 0.122 | **0.045** |
| ***Disease indicators*** *^a^* |  |  |  |  |  |  |  |  |
| Rheumatoid Factor (UI/mL) | 0.287 | **0.039** | − 0.287 | **0.039** | − | − | − | − |

^a^ Rheumatoid arthritis with insulin resistance group n = 58. P values were calculated using rho Spearman correlation test, (P < 0.05 was significant). Bold numbers show significate correlations. Abbreviations: BMI: body mass index; HOMA-IR: homeostasis model assessment of insulin resistance; QUICKI: quantitative insulin sensitivity check index; HOMA-B: homeostatic model assessment of β-cell; DI: basal disposition index. HDLc, LDLc and VLDLc (high, low and very low-density lipoproteins cholesterol, respectively); Apo: apolipoprotein; CRP: C-reactive protein.
