# Supplementary figures and images for: CCR2/CCL2 and CMKLR1/RvE1 chemokines system levels are associated with insulin resistance in rheumatoid arthritis
Source: PLoS One. 2021 Jan 28;16(1):e0246054. doi: 10.1371/journal.pone.0246054 (PMC7842933; doi:10.1371/journal.pone.0246054)

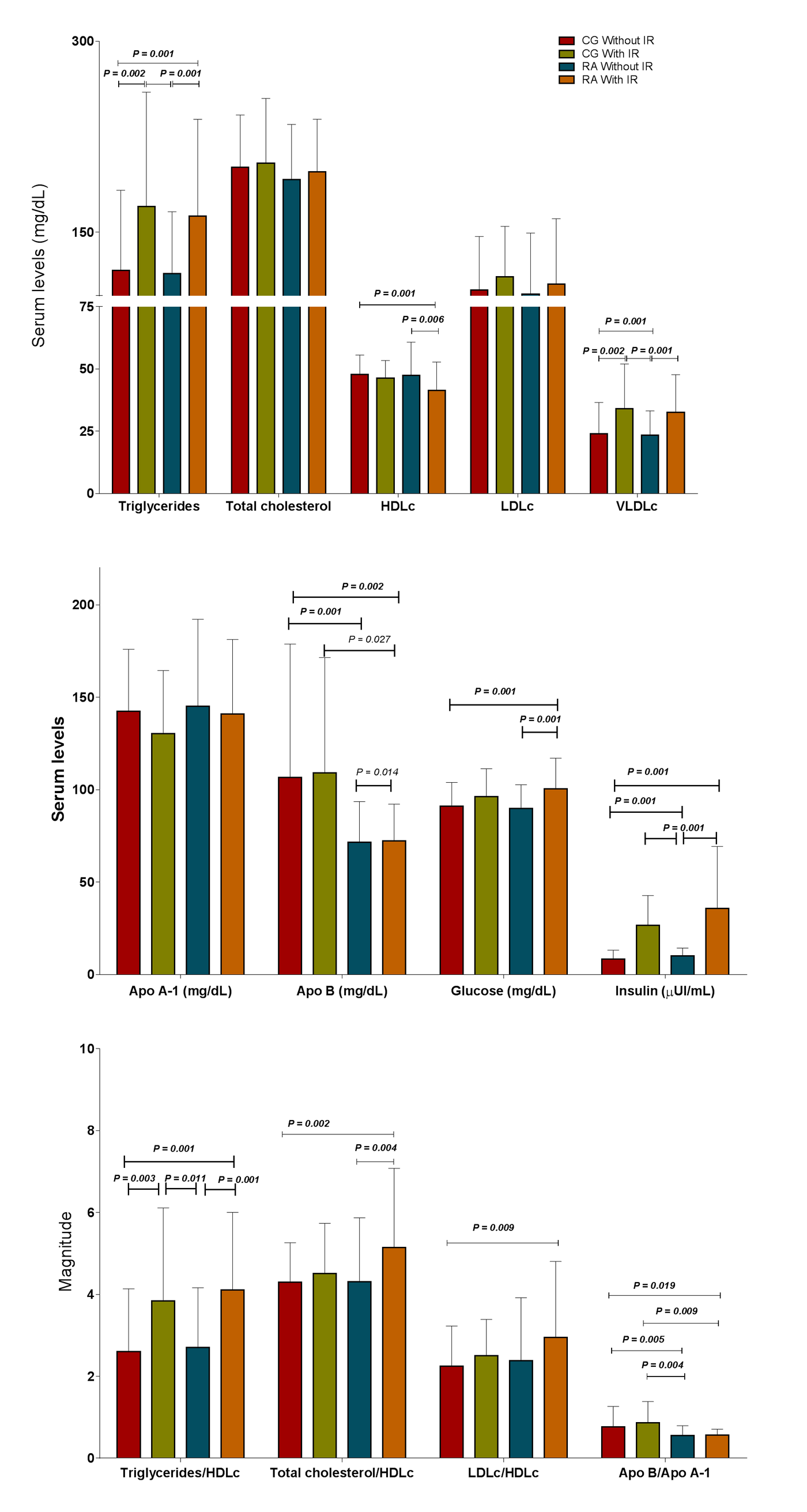

Supplement: S1 Fig — A) Lipids profile, B) Metabolic markers, C) Cholesterol ratios. The results are show in x¯ ± SD. Kruskal-Wallis H test, (P < 0.05 was significant). Bold lines (—) show differences between groups. Abbreviations: RA: rheumatoid arthritis; IR: insulin resistance; HDLc, LDLc and VLDLc (high, low and very low-density lipoproteins cholesterol, respectively); Apo: apolipoprotein. (TIF) [file pone.0246054.s003.tif]

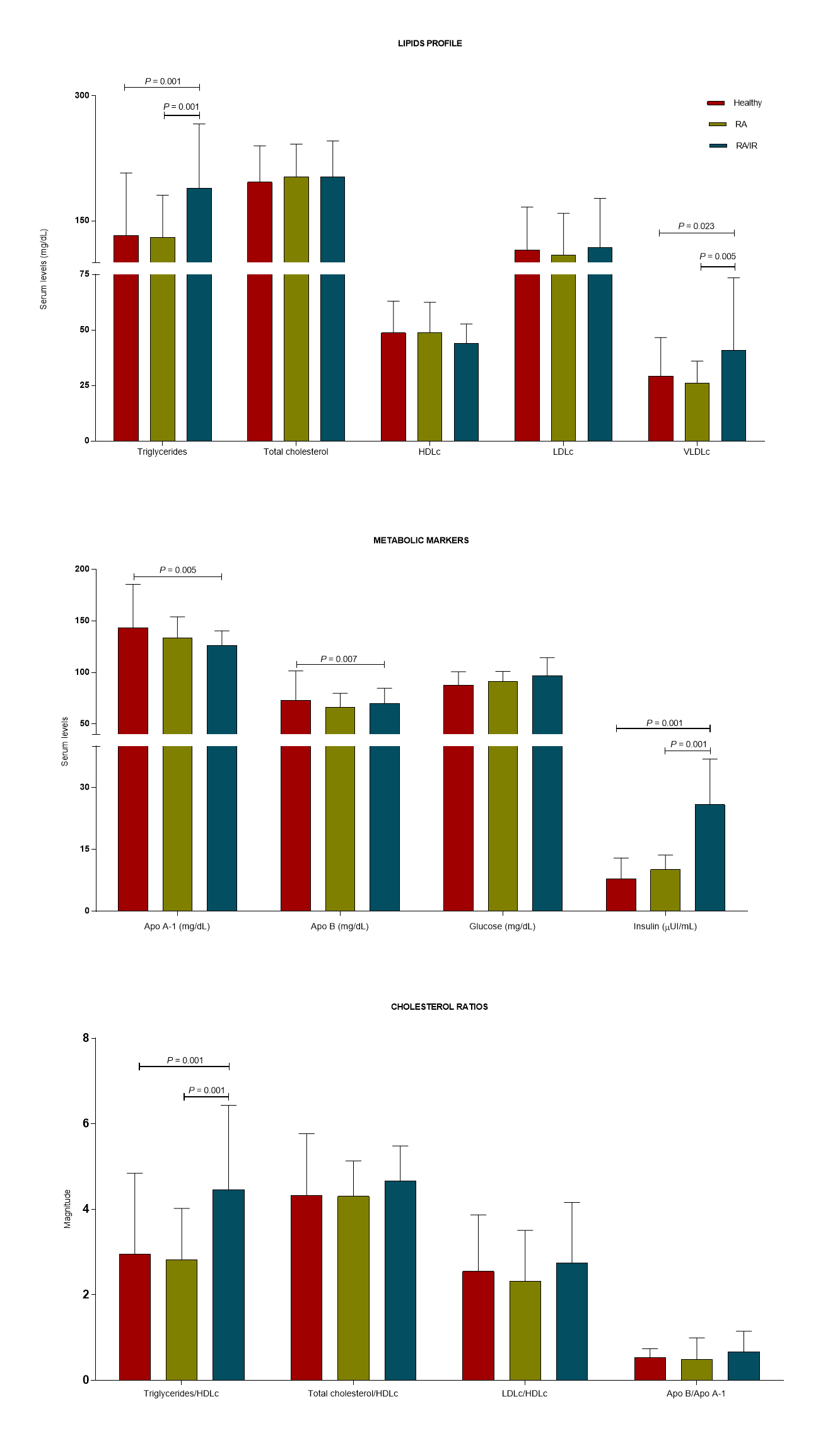

Supplement: S1 Data — (TIF) [file pone.0246054.s004.tif]
