# Supplementary material for: CCR2/CCL2 and CMKLR1/RvE1 chemokines system levels are associated with insulin resistance in rheumatoid arthritis
Source: PLoS One. 2021 Jan 28;16(1):e0246054. doi: 10.1371/journal.pone.0246054 (PMC7842933; doi:10.1371/journal.pone.0246054)
Supplement: S2 Data — (DOCX) [file pone.0246054.s005.docx]

S2 Data. IR *status* correlations with adiposity in study group.

| Measurements | HOMA-IR | | QUICKI | | HOMA-B | | DI | |
| --- | --- | --- | --- | --- | --- | --- | --- | --- |
|  | *rho* | *P* | *rho* | *P* | *rho* | *P* | *rho* | *P* |
| *Storage of body fat mass* | | | | | | | | |
| Body weight (kg) | 0.255 | 0.002 | − 0.201 | 0.002 | 0.300 | 0.001 | - | - |
| Total body fat mass (%) | 0.291 | 0.001 | − 0.253 | 0.002 | 0.311 | 0.001 | - | - |
| Total body fat mass (kg) | 0.294 | 0.001 | − 0.253 | 0.002 | 0.318 | 0.001 | - | - |
| *Distribution of body fat mass* | | | | | | | | |
| Trunk fat mass (kg) | 0.261 | 0.001 | − 0.215 | 0.009 | 0.180 | 0.029 | - | - |
| Trunk fat mass (%) | - | - | - | - | 0.291 | 0.001 | 0.185 | 0.027 |
| Upper limbs fat mass (%) | 0.350 | 0.001 | − 0.362 | 0.001 | 0.349 | 0.001 | - | - |
| Upper limbs fat mass (kg) | 0.326 | 0.001 | − 0.290 | 0.001 | 0.345 | 0.001 | - | - |
| Lower limbs fat mass (%) | − 0.165 | 0.043 | - | - | − 0.241 | 0.003 | - | - |
| Lower limbs fat mass (kg) | 0.315 | 0.001 | − 0.281 | 0.001 | 0.314 | 0.001 | - | - |
| *Body dimensions (cm)* | | | | | | | | |
| Waist circumference (cm) | 0.286 | 0.001 | − 0.271 | 0.001 | 0.211 | 0.011 | - | - |
| Hip circumference (cm) | 0.251 | 0.002 | − 0.206 | 0.012 | 0.310 | 0.001 | - | - |
| Coronal diameter (cm) | 0.220 | 0.007 | - | - | - | - | - | - |
| *Obesity indexes* | | | | | | | | |
| BMI (kg/m^2^) | 0.353 | 0.001 | − 0.331 | 0.001 | 0.314 | 0.001 | - | - |
| Body fat ratio | 0.303 | 0.001 | − 0.265 | 0.001 | 0.312 | 0.001 | - | - |
| Waist to height ratio | 0.301 | 0.001 | − 0.298 | 0.001 | 0.201 | 0.001 | - | - |
| Waist-hip ratio | 0.193 | 0.017 | − 0.202 | 0.015 | - | - | - | - |
| Visceral area (cm^2^) | 0.283 | 0.001 | − 0.269 | 0.001 | 0.251 | 0.003 | - | - |
| Abdominal volume index | 0.284 | 0.001 | - | - | 0.215 | 0.009 | - | - |

Notes: *rho* (Spearman correlation test).

Abbreviations: BMI: body mass index; HOMA-IR: homeostasis model assessment of insulin resistance; QUICKI: quantitative insulin sensitivity check index; HOMA-B: homeostatic model assessment of β-cell; DI: basal disposition index.
