# Supplementary material for: CCR2/CCL2 and CMKLR1/RvE1 chemokines system levels are associated with insulin resistance in rheumatoid arthritis
Source: PLoS One. 2021 Jan 28;16(1):e0246054. doi: 10.1371/journal.pone.0246054 (PMC7842933; doi:10.1371/journal.pone.0246054)
Supplement: S3 Data — (DOCX) [file pone.0246054.s006.docx]

S3 Data. IR status correlations with immuno-metabolic markers in study group.

| Measurements | HOMA-IR | | QUIKI | | HOMA-B | | DI | |
| --- | --- | --- | --- | --- | --- | --- | --- | --- |
|  | *rho* | *P* | *rho* | *P* | *rho* | *P* | *rho* | *P* |
| *Lipid profile* | | | | | | | | |
| Triglycerides (mg/dL) | 0.277 | 0.001 | − 0.244 | 0.003 | - | - | - | - |
| VLDLc (mg/dL) | 0.215 | 0.008 | − 0.195 | 0.019 | - | - | − 0.226 | 0.007 |
| Triglycerides/HDL | 0.306 | 0.001 | − 0.285 | 0.001 | 0.226 | 0.006 | - | - |
| HDLc (mg/dL) | **-** | **-** | **-** | **-** | − 0.243 | 0.003 | − 0.166 | 0.006 |
| Apo A-1 | - | - | - | - | − 0.279 | 0.002 | - | - |
| LDLc (mg/dL) | - | - | - | - | - | - | 0.195 | 0.020 |
| LDLc/HDLc | - | - | - | - | - | - | 0.280 | 0.001 |
| *Inflammation markers* | | | | | | | | |
| CRP (mg/L) | 0.266 | 0.001 | − 0.208 | 0.012 | 0.200 | 0.015 | - | - |
| *Disease indicators** | | | | | | | | |
| RF (UI/mL) | 0.307 | 0.008 | − 0.303 | 0.009 | 0.270 | 0.020 | - | - |
| ACPA (UI/mL) | 0.267 | 0.024 | − 0.235 | 0.049 | - | - | - | - |
| DAS28-CRP | − 0.269 | 0.021 | 0.274 | 0.019 | - | - | - | - |
| CDAI | − 0.268 | 0.021 | 0.261 | 0.026 | - | - | - | - |

Notes: *rho* (Spearman correlation test). *RA/IR group.

Abbreviations: HOMA-IR: homeostasis model assessment of insulin resistance; QUICKI: quantitative insulin sensitivity check index; HOMA-B: homeostatic model assessment of β-cell; DI: basal disposition index; VLDLc: very low density lipoprotein of cholesterol; HDLc: high density lipoprotein cholesterol; LDLc: low density lipoprotein cholesterol; Apo: apolipoprotein; CRP: C-reactive protein; RF: rheumatoid factor; ACPA: anticyclic citrullinate peptide antibody; DAS28-CRP: disease activity score on 28 joints with C-reactive protein; CDAI: clinical disease activity index.
